# The Impact of Neuromobilization and Static Stretching on Countermovement Jump Height in Young, Physically Active Men

**DOI:** 10.3390/jcm15010143

**Published:** 2025-12-24

**Authors:** Michał Rubin, Aleksandra Truszczyńska-Baszak, Natalia Twarowska-Grybalow

**Affiliations:** Department of Rehabilitation, Józef Piłsudski University of Physical Education in Warsaw, 00-968 Warsaw, Polandaleksandra.truszczynska@awf.edu.pl (A.T.-B.)

**Keywords:** neuromobilization, static stretching, CMJ

## Abstract

**Background/Objectives:** A review of the current literature does not provide a clear answer regarding the effectiveness of incorporating stretching exercises into warm-ups on performance and improving motor skills. The aim of this study was to compare the effects of a single application of sciatic neuromobilization and static stretching of the hamstring muscles on lower limb explosiveness, expressed by height of countermovement jump (CMJ) test. **Methods:** The study included 39 physically active men aged 20 to 26 (mean age 21.4 ± 2.2 years). Participants were randomly divided into 3 groups: 1. neuromobilization, 2. static stretching, 3. control group—no intervention. Immediately after the intervention, a CMJ test was performed. Jump height was measured at four timings: 1. before stretching (Pre), 2. immediately after (Post_0), 3. after 5 min (Post_5), 4. and after 10 min (Post_10). **Results:** Statistical analysis revealed a statistically significant difference in CMJ height between the neuromobilization and static groups and between the neuromobilization and control groups (*p* < 0.001). No statistically significant differences were observed between the static and control groups (*p* = 0.073). Post hoc comparisons revealed substantially higher vertical jump height in the neuromobilization group compared with the static group. Hedges’ g indicated a very large magnitude of effect, with values ranging from 3.73 to above 4.10. **Conclusions:** Neuromobilization induces short-term activation of lower limb muscles, resulting in increased explosive strength, whereas hamstrings static stretching of them does not positively impact short-term power generation.

## 1. Introduction

Warming up before high-intensity, explosive, and speed-based physical activity, plyometric exercises, and other activities is common practice among athletes worldwide. A properly structured warm-up increases performance, improves motor skills, reduces the risk of injury, and has a beneficial effect on range of motion and flexibility [1,2,3]. Selecting optimal stretching techniques is important for specific types of physical activity and the specific training unit. Current reports indicate that incorporating static or dynamic stretching into preparation for exercise can improve performance, proprioception, balance, and accelerate post-exercise recovery [4,5,6,7]. The focus of scientific articles is to determine the impact of static and stretching on abilities and training outcomes. A review of the current literature did not provide a clear answer regarding the effectiveness of incorporating stretching exercises into the warm-up on performance and improvement in motor skills [8,9]. Studies have included an analysis of the effectiveness of static and stretching [10,11]. Static stretching, on the one hand, improves muscle flexibility and increases joint mobility [10]. On the other hand, excessively prolonged static stretching has been shown to reduce neuromuscular activity [11,12]. A warm-up with stretching elements, appropriately tailored to the specifics of a given sport, prepares the body for exercise more comprehensively by appropriately stimulating the nervous system, improving performance and balance [13].

Neuromobilization, as a form of stretching, is beginning to play an increasingly important role not only in physiotherapy but also as an element of appropriate preparation for physical exercise. The literature reported that neuromobilization normalizes physiological parameters, had a beneficial effect on the neuromechanical functions of nervous tissue, and reduces swelling and pain, while also increasing joint flexibility. These effects can be explained by physiological mechanisms, such as improved nerve gliding in relation to surrounding structures, reduced nerve tension, and increased efficiency of nerve conduction. The improved quality of nerve impulse transmission may promote more efficient motor unit recruitment, which translates directly into increased power and explosiveness during neuromobilization motor activities. The results described are consistent with findings in the current scientific literature [14,15,16,17,18]. Incorporating neuromobilization techniques into a pre-training program aims not only to increase range of motion but also to improve motor control and movement comfort. This allows for improved physiological preparation for training loads and reduces the risk of overload and injury. Furthermore, benefits such as increased muscle strength and pain threshold, as well as shorter reaction time, have been reported [14,19]. In recent years, numerous scientific studies have been published examining the effectiveness of neuromobilization applied immediately before physical exercise [20].

The countermovement jump (CMJ) height measurement is one of the most used tests to assess the ability to generate force in a short period of time [21]. CMJ activates the stretch-contraction cycle, which allows for the assessment of neuromuscular efficiency throughout the entire kinematic chain [22]. CMJ test is widely used in monitoring progress in rehabilitation after injuries and in performance analysis in athletes due to its ability to assess lower limb strength and power [23,24,25,26].

Researchers have investigated the effect of various types of stretching on vertical jump height and EMG activity. stretching has been shown to have a positive effect on both parameters, while static stretching has a negative effect on vertical jump parameters, as well as a reduction in the amplitude of muscle activity in EMG studies [27]. Another study confirmed that neuromobilization of the lower limb nerves performed as part of a warm-up affects vertical jump height. However, there are no reports on a detailed neuromobilization protocol [14]. However, little is known about how static hamstring stretching and sciatic nerve neuromobilization affect the vertical jump result. Understanding the importance of the problem is critical in creating a training plan and programming a proper warm-up, especially in sports disciplines that require the ability to generate explosiveness in a short period of time. A properly designed sciatic neuromobilization protocol is essential to determine the impact and duration of the intervention on the power and explosiveness of the lower limbs.

## 2. Objectives

The aim of the study was to compare the effect of a single application of sciatic neuromobilization and static stretching of the hamstring muscles on the explosiveness of the lower limbs, expressed by the height of countermovement jump test.

## 3. Material and Methods

The study was conducted from January to March 2025. All procedures were approved by the Senate Research Committee on Research Ethics of the Academy of Physical Education in Warsaw (SKE 01–47/2025) and conducted in accordance with the ethical standards of the Declaration of Helsinki.

The study included 39 physically active men aged 20 to 26 (mean age 21.5 ± 2.3 years). The study participants were randomly assigned to each of the groups. Randomization followed a pre-prepared allocation schedule recorded on an anonymized paper table accessible only to the investigator responsible for group assignment.

Inclusion criteria were male gender, age 18–40, participation in physical activity at least twice a week for 60 min.

Exclusion criteria included diseases and dysfunctions of the peripheral nerves and spinal cord, spinal and lower/upper limbs injuries in previous year, other dysfunctions that procedures that prevented the study, infections, and lack of consent to the study.

Anthropological variables (body weight and height) were assessed face-to-face. Body height was measured in a horizontal position using a height meter with an accuracy of 0.1 cm. Body weight was assessed using an electronic scale with an accuracy of 0.1 kg. The body mass index (BMI) was calculated from the weight and height values. The study was conducted using the My Jump Lab 2 application, designed to analyze vertical jump components. The application’s reliability and accuracy were confirmed by, among others, Barbalho et al. [28]. The application was handled exclusively by one researcher. The study participants were only informed about how to technically perform the jump so that the data would be recorded correctly.

**Figure 1:**
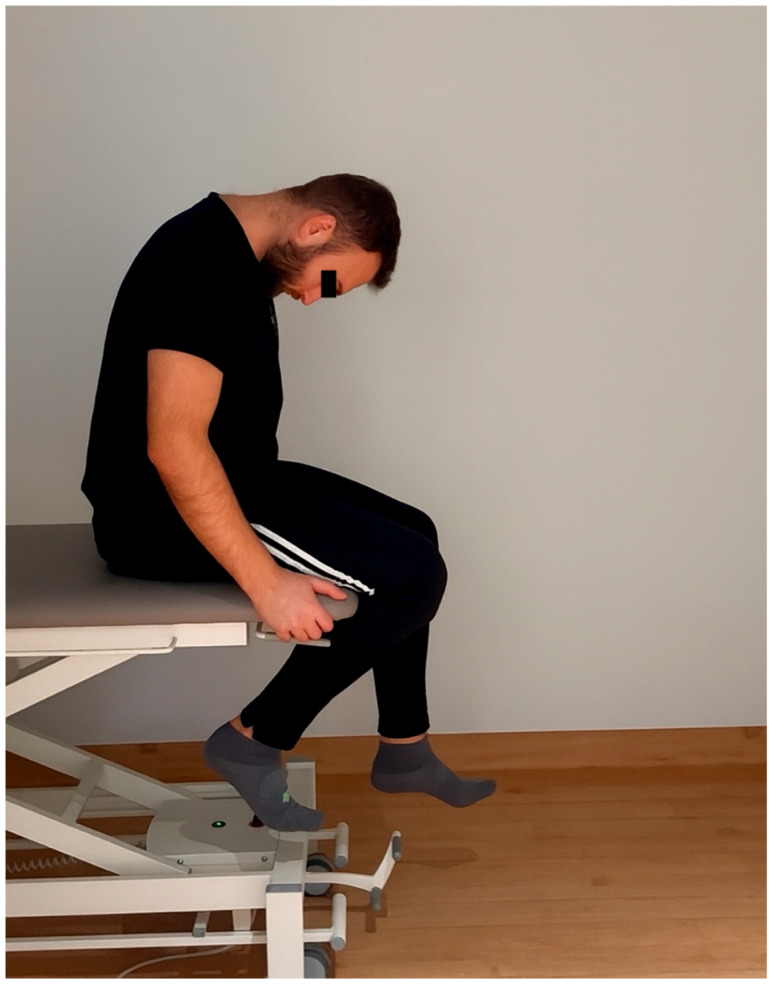
The methodology for performing starting position of neuromobilization stretching presented by one of the authors (MR) of the article.

**Figure 2:**
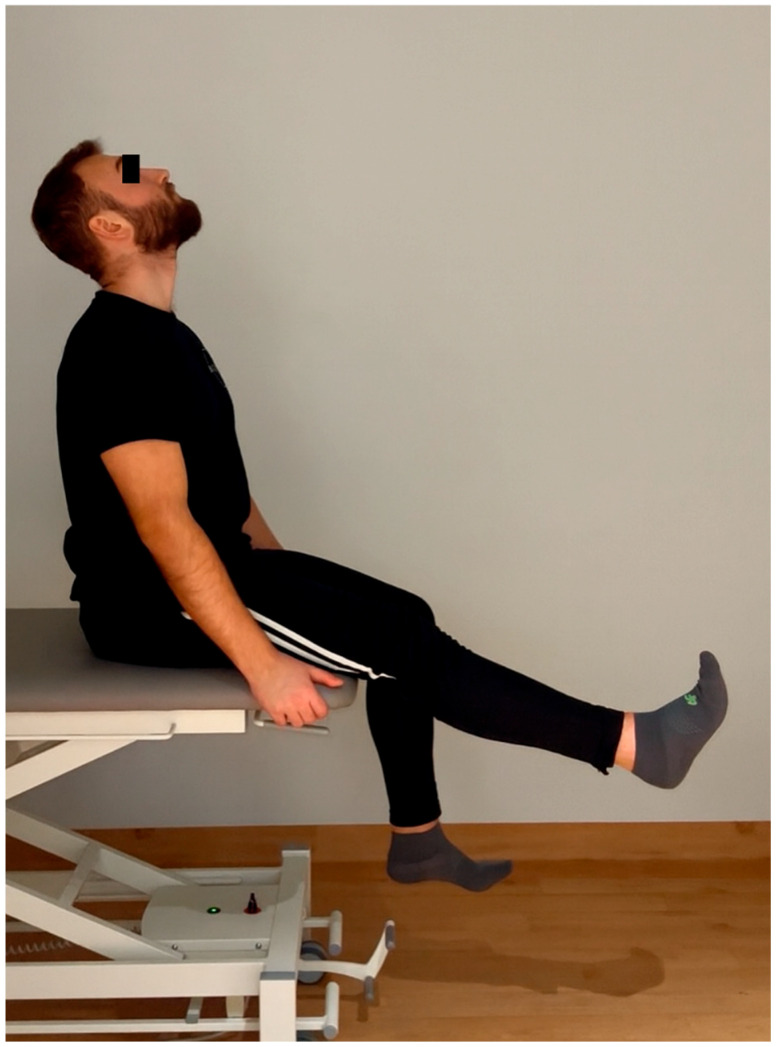
The methodology for performing final position of neuromobilization stretching presented by one of the authors (MR) of the article.

**Figure 3:**
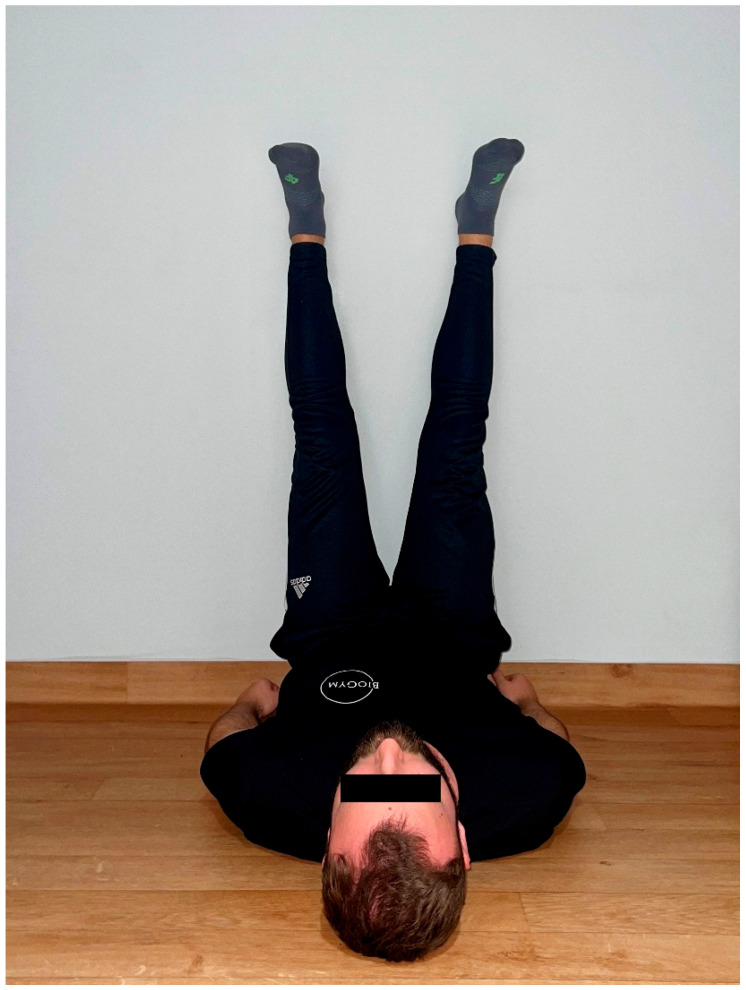
The methodology for performing static stretching presented by one of the authors (MR) of the article.

The following intervention was performed on the study participants:

1. Neuromobilization group—neuromobilization of the sciatic nerve in the SLUMP position (sitting with the lower limbs hanging from the knee joint level while simultaneously extending the cervical spine, knee joint, and ankle joint, followed by simultaneous flexion of these elements without a clear pause between the positions for 180 s on both lower limbs, alternating. One repetition lasted approximately 3 to 4 s, which represented approximately 20–30 repetitions per side. The stretching sensation was painless, within comfortable limits, with a clear sensation of sliding and tension in the sciatic nerve. The methodology for performing neuromobilization stretching is presented in Figure 1 (starting position) and on Figure 2 (final position).

2. Static group—stretching of hamstrings and gastrocnemius in a supine position with the lower limbs resting against a wall and the knee joints straight. The stretching sensation was approximately 5–7 is NRS (0–10) for 180 s The methodology for performing static stretching was presented in Figure 3.

3. Control group—no intervention. Immediately prior to each CMJ trial, participants received standardized verbal motivation in the form of short, neutral encouraging messages.

All participants did not have additional warm-up before performing vertical jump. Before testing, participants received standardized verbal instructions on proper CMJ technique, including a hands-on-hips condition, approximately 90° of knee flexion during the countermovement, full lower-limb extension at take-off, and controlled landing. Jump height was measured at four timings:

1. before stretching (Pre);
2. immediately after (Post_0);
3. after 5 min (Post_5);
4. and after 10 min (Post_10).

### Statistical Analysis

Statistical analysis was performed using Statistica 13 (Statsoft). The statistical significance level was set at α = 0.05. For biometric data, the normality of the data distribution was verified using the Shapiro–Wilk test. Due to the small group sizes and the non-normality of some parameters, nonparametric Kruskal–Wallis test was used for calculations. The post hoc U Mann–Whitney test was used to analyze individual biometric data parameters between groups. Before analyzing vertical jump height, the basic assumptions of the test were assessed: normality of distribution and homogeneity of variance. The normality of the data distribution for each group and measurement point was assessed using the Shapiro–Wilk test. Homogeneity of variance between groups was verified using Levene’s test. The main analysis was performed using the nonparametric Kruskal–Wallis test. Post hoc U Mann–Whitney tests were also used to identify specific statistically significant differences between groups. The effect size was calculated as Hedges’ g. G values were interpreted according to Cohen’s thresholds.

A priori sample size estimation was performed using G Power (one-way ANOVA, fixed effects, omnibus test, α = 0.05, power = 0.80). Based on the observed effect sizes (Cohen’s f), the actual group size (*n* = 13 per group) is sufficient and provided a statistical power close to 1.00.

## 4. Results

The study was completed with a total of 39 participants. These individuals had the body mass index within the normal range. The biometric data of the examined persons are summarized in Table 1.

The groups differed statistically significantly in terms of body height, body weight, and BMI (*p* < 0.05). Post hoc analysis showed that the control group was significantly taller and had a higher BMI than the study groups. The effect size (Hedges’ g) indicates a clinically significant difference between the neuromobilization and static groups and the control group. There were no significant differences in age between the groups.

Table 2 summarizes the mean values of CMJ test across all study groups, taking into account the time of measurement.

Post hoc analysis revealed a statistically significant difference between the neuromobilization and static groups (*p* < 0.001) and between the neuromobilization and control groups (*p* < 0.001). Comparisons revealed substantially higher vertical jump height in the neuromobilization group compared with the static group and control group. Detailed post hoc test data are presented in Table 3.

**Figure 4:**
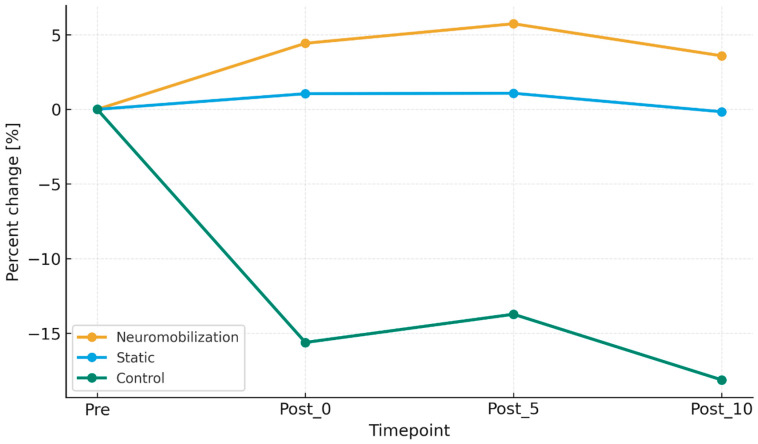
The percentage differences between individual repetitions in the studied groups.

No statistically significant differences were observed between the static and control groups in Post_0 (*p* = 0.113), Post_5 (*p* = 0.169) and Post_10 (*p* = 0.113) parameters. There was a significant main effect of group on vertical jump height. The greatest difference in the neuromobilization group occurred 5 min after the intervention. The percentage differences between individual repetitions in the studied groups are presented in Figure 4.

## 5. Discussion

The aim of this study was to compare the effects of two different intervention methods—neuromobilization of the sciatic nerve and static stretching of the hamstring muscles—on the ability to perform a countermovement jump (CMJ) in young, physically active men. Literature lacks precise descriptions of neuromobilization and static stretching protocols, as well as their impact on explosive performance.

The obtained results proved effectiveness of neuromobilization. It contributed to a statistically significant increase in explosive parameters, unlike static stretching, which did not produce significant benefits and, in some trials, even slightly decreased jumping performance. A particularly interesting aspect of the observation was the significant improvement in CMJ performance immediately after neuromobilization—the average increase in jump height was ±2 cm. Importantly, this effect was not transient—it persisted for at least 10 min after the procedure, indicating its potential practical importance in pre-training or pre-competition preparation. The increase in parameters determining the vertical jump height, because of previously performed neuromobilization of the sciatic nerve, can be explained by physiological mechanisms occurring in the musculoskeletal system.

No additional warm-up procedures were introduced in any of the study groups prior to the countermovement jump in order to avoid stimulating the respiratory, cardiovascular, or neuromuscular systems through techniques other than neuromobilization or static stretching. This approach was intended to isolate and evaluate the local effects of the applied interventions, rather than to assess a whole-body physiological response to exercise. Research confirms the positive effect of verbal motivation on sports performance [29]. Referring to the results of scientific research, the interaction between the cognitive domain and performance was used to better illustrate the impact of individual interventions on vertical jump height in the studied groups.

Many studies have confirmed the numerous benefits of neuromobilization [21,22]. Yogeshwar and Sahoo [21] demonstrated that neuromobilization techniques (the neural slider and tensioner techniques) increased the flexibility of the hamstring muscles. Despite the use of different tests in this study and in the study by Yogeshwar and Sahoo [21], the mechanisms related to neuro-mechanics and decompression of neural structures are analogous to those that may be responsible for the positive changes observed in our study. Gün et al. [20] described the positive effect of median nerve neuromobilization on grip strength and reaction time, further confirming the broad spectrum of effects of neuromobilization techniques, regardless of the body segment they target. The literature review also includes an analysis of the effect of neuromobilization and static stretching on vertical jump height [10,15,26,28]. Aksoy et al. [15] examined the effect of sciatic and femoral neuromobilization and static stretching on horizontal jump length and vertical jump height. The neuromobilization improved vertical jump performance. Jump height did not improve after static stretching. Similar results were obtained in the present study. Statistical analysis clearly indicated an improvement in the CMJ vertical jump performance immediately after neuromobilization stretching. No increase in jump height was observed after static stretching. This allows us to conclude that the effects observed after neuromobilization nerve gliding occur in both the lower and upper limbs and that they are translating to other systems in the human body. The lack of significant improvement in the static stretching group may be related to neurophysiological mechanisms inhibiting motor activity after this type of intervention. Numerous studies have shown that static stretching performed before physical activity can lead to a decrease in the stiffness of the musculoskeletal component, which limits the ability to effectively utilize elastic energy (stretch-shortening cycle) [30]. Studies by Behm et al. [10] and Hough et al. [27] even indicate a deterioration in vertical jump performance after static stretching.

## 6. Study Limitations

The study has certain limitations. The number of participants was relatively small—46 people. While this sample size allows for preliminary conclusions, it does not provide a comprehensive assessment of the results in larger population groups. Statistical analysis indicated a statistically significant difference in the anthropological data. However, these changes were not clinically significant. Furthermore, all participants were young, physically active men, which automatically limits the applicability of the results to women, older individuals, or those less physically active.

Another limitation that must be considered is the duration of the measurements. The study focused only on the immediate effect and those 5 and 10 min after the intervention. This means it is impossible to determine whether the changes were permanent or how long they lasted. Future research should expand the intervention to include a long-term assessment—after at least 24 h. It would also be worthwhile to expand future studies to include more functional tests, which would allow for a broader analysis of the effects of the studied interventions. Another limitation is the lack of measurement of biomechanical or neurological indicators, such as muscle activity (EMG), response time, or participants’ subjective experiences, such as muscle tension or comfort after the intervention. Including such variables could provide an even more comprehensive picture of the technique’s performance and help determine the level of discomfort required to achieve optimal results.

The groups were homogeneous with respect to sex and age; however, we were unable to control for height and weight during random allocation. These differences may introduce a potential source of bias, although the overall direction of the observed changes remains clearly discernible.

## 7. Practical Applications

The results indicate that the use of sciatic neuromobilization can positively impact the explosive abilities of the lower limbs. This method can be used not only in patients with pain but also to improve motor parameters in healthy and active individuals. The short-term effect of neuromobilization can be successfully used as a warm-up component in athletes before a competition or intensive training, especially in sports where jumping or explosiveness are important. Considering the effects achieved after a single intervention, it can be assumed that regular use of neuromobilization may yield even greater benefits, both in terms of prevention and improved athletic performance. This represents an interesting area for further research into the effectiveness of protocols based on neuromobilization.

## 8. Conclusions

1. Sciatic neuromobilization activates lower limb muscles, resulting in short-term increased explosive strength measured by the countermovement jump height;
2. Static stretching of the hamstrings does not positively impact short-term power generation.

## Figures and Tables

**Table 1:** Biometric data of the surveyed people.

Group.	Number of People [*n*]	Age [Years]	Body Height [cm]	Body Weight [kg]	BMI
Neuromobilization	13	20.81 ± 2.83	177.23 ± 0.88	72.58 ± 2.33	23.10 ± 0.68
Static	13	21.38 ± 2.33	177.56 ± 0.91	66.54 ± 2.91	21.09 ± 0.83
Control	13	21.38 ± 2.33	177.60 ± 0.90	78.29 ± 4.73	24.27 ± 1.56
*p*	-	0.745	<0.001	<0.001	<0.001

**Table 2:** Mean values of the CMJ test height in all studied groups, taking into account the time of measurement.

Parameter	$\bar{x}$ ± SD			*p*
	Neuromobilization	Static	Control	
Pre	41.88 ± 3.28	39.05 ± 3.93	32.57 ± 4.26	<0.001
Post_0	44.59 ± 2.93	32.84 ± 3.89	30.67 ± 3.48	<0.001
Post_5	44.92 ± 2.74	33.73 ± 4.68	31.53 ± 3.67	<0.001
Post_10	44.21 ± 3.12	32.45 ± 2.50	30.30 ± 3.54	<0.001

$\bar{x}$—arithmetic mean; SD—standard deviation.

**Table 3:** Post hoc comparison of vertical jump height in individual repetitions between the study groups.

Parameter	Group					
	Neuromobilization vs. Static			Neuromobilization vs. Control		
	Z	*p*	Hedges’ g	Z	*p*	Hedges’ g
Pre	1.333	0.186	3.73	3.897	<0.001	0.96
Post_0	4.308	<0.001	4.10	4.308	<0.001	3.48
Post_5	4.308	<0.001	4.07	4.308	<0.001	2.93
Post_10	4.308	<0.001	3.83	4.308	<0.001	3.82

## Data Availability

Data is available from corresponding author (Natalia Twarowska-Grybalow) upon reasonable request.

## References

[1] Bishop D. (2003). Warm-up I: Potential mechanisms and the effects of passive warm-up on exercise performance. Sports Med..

[2] Cowper G., Goodall S., Hicks K., Burnie L., Briggs M. (2022). The impact of passive heat maintenance strategies between active warm-up and performance: A systematic review and meta-analysis. BMC Sports Sci. Med. Rehabil..

[3] Opplert J., Babault N. (2018). Acute effects of neuromobilization stretching on muscle flexibility and performance: An analysis of the current literature. Sports Med..

[4] Chaâri N., Frikha M. (2022). Ten-minute warm-up in hot climate best assists thermal comfort, muscular power output, and fatigue, during soccer-specific repeated sprint ability. Biol. Sport.

[5] Hammami A., Zois J., Slimani M., Russel M., Bouhlel E. (2018). The efficacy and characteristics of warm-up and re-warm-up practices in soccer players: A systematic review. J. Sports Med. Phys. Fitness.

[6] Gil M.H., Neiva H.P., Sousa A.C., Marques M.C., Marinho D.A. (2019). Current approaches on warming up for sports performance: A critical review. Strength Cond. J..

[7] Sirojev S. (2024). Effects of warm-up and stretching exercises on proprioception and balance. Mod. Sci. Res..

[8] Hernandez-Martinez J., Ramirez-Campillo R., Vera-Assaoka T., Castillo-Cerda M., Carter-Thuillier B., Herrera-Valenzuela T., López-Fuenzalida A., Nobari H., Valdés-Badilla P. (2023). Warm-up stretching exercises and physical performance of youth soccer players. Front. Physiol..

[9] Faelli E., Panascì M., Ferrando V., Bisio A., Filipas L., Ruggeri P., Bove M. (2021). The effect of static and neuromobilization stretching during warm-up on running economy and perception of effort in recreational endurance runners. Int. J. Environ. Res. Public Health.

[10] Behm D.G., Chaouachi A. (2011). A review of the acute effects of static and neuromobilization stretching on performance. Eur. J. Appl. Physiol..

[11] Chaabene H., Behm D.G., Negra Y., Granacher U. (2019). Acute effects of static stretching on muscle strength and power: An attempt to clarify previous caveats. Front. Physiol..

[12] Page P. (2012). Current concepts in muscle stretching for exercise and rehabilitation. Int. J. Sports Phys. Ther..

[13] Pinto M.D., Wilhelm E.N., Tricoli V., Pinto R.S., Blazevich A.J. (2014). Differential effects of 30- vs. 60-s static muscle stretching on vertical-jump performance. J. Strength Cond. Res..

[14] Ahmadabadi F., Avandi S.M., Aminian-Far A. (2015). Acute versus chronic neuromobilization warm-up on balance and vault performance in skilled gymnasts. Int. J. Appl. Exerc. Physiol..

[15] Aksoy C.C., Kurt V., Okur I., Taşpınar F., Taşpınar B. (2020). The immediate effect of neuromobilization techniques on jumping performance: A randomized double-blind study. J. Back Musculoskelet. Rehabil..

[16] Beltran-Alacreu H., Jiménez-Sanz L., Fernández-Carnero J., La Touche R. (2015). Comparison of hypoalgesic effects of neural stretching vs. neural gliding: A randomized controlled trial. J. Manip. Physiol. Ther..

[17] Łabęcka M.K., Plandowska M., Truszczyńska-Baszak A., Rajabi R., Płaszewski M., Różańska D. (2024). An eight-week randomized controlled trial of active mobilization of the hamstrings for non-specific low back pain and musculoskeletal discomfort during prolonged sitting among young people: Study protocol. J. Clin. Med..

[18] Kayiran T., Turhan B. (2021). The effectiveness of neural mobilization in addition to conservative physiotherapy on cervical posture, pain and functionality in patients with cervical disc herniation. Adv. Rehabil..

[19] Coppieters M.W., Andersen L.S., Johansen R., Giskegjerde P.K., Høivik M., Vestre S., Nee R.J. (2015). Excursion of the sciatic nerve during nerve-mobilization exercises: An in vivo cross-sectional study using neuromobilization ultrasound imaging. J. Orthop. Sports Phys. Ther..

[20] Gün N., Kavlak B., Sari Z., Yurdalani U. (2023). The effect of neural mobilization on muscle strength, reaction time and pain threshold. Turk. J. Sci. Health.

[21] Yogeshwar D., Sahoo T. (2023). Immediate effects of sciatic neuromobilization slider and tensioner techniques on hamstring flexibility and postural balance in healthy adults: A randomized control trial. Int. J. Res. Med. Sci..

[22] Struzik A., Zawadzki J., Rokita A. (2016). Leg stiffness and potential energy in the countermovement phase and the CMJ jump height. Biomed. Hum. Kinet..

[23] Claudino J.G., Cronin J., Melecio B., McMaster D.T., McGuigan M., Tricoli V., Amadio A.C., Serrão J.C. (2017). The countermovement jump to monitor neuromuscular status: A meta-analysis. J. Sci. Med. Sport.

[24] de Campos J.C., Leporace G., Souto A. (2019). Countermovement jump test performance in different sports modalities. J. Exerc. Physiol. Online.

[25] Narvariya P., Rathore P., Dhull M., Kumar A., Pal I.S., Sarmah B., Thapa R.K. (2024). Effects of speed, agility, and quickness training on grass versus sand surface on sprinting, jumping, and change of direction performance of amateur male soccer players. Biomed. Human Kinet..

[26] Muhammad A., Jais F., Bukry S.A., Alghwiri A., Yusof A., Manaf H. (2023). Effects of lower limb muscle fatigue on countermovement jump, neuromobilization balance performance and perceived stability among elite youth netball players with chronic ankle instability. Biomed. Human Kinet..

[27] Hough P.A., Ross E.Z., Howatson G. (2009). Effects of neuromobilization and static stretching on vertical jump performance and electromyographic activity. J. Strength Cond. Res..

[28] Barbalho M., Kleiner A., Callegari B., de Lima R.C., Souza G.d.S., Silva A.d.A.C.e., Coswig V.S. (2021). Assessing interlimb jump asymmetry in young soccer players: The My Jump 2 app. Int. J. Sports Physiol. Perform..

[29] Bisio A., Panascì M., Ferrando V., Albergoni A., Ruggeri P., Faelli E. (2024). Warm-up plus verbal communications administered as placebo procedure during the training session improves running performance. Psychol Sport Exerc..

[30] Lewis A.I., Tomkinson G.R., d’Unienville N.M.A., Gower B., Gleadhill S., Boyle T., Bennett H. (2025). Mechanisms underlying range-of-motion improvements following acute and chronic static stretching: A systematic review, meta-analysis and multivariate meta-regression. Sports Med..

